# Parasitic Intestinal Protists of Zoonotic Relevance Detected in Pigs by Metabarcoding and Real-Time PCR

**DOI:** 10.3390/microorganisms9061189

**Published:** 2021-05-31

**Authors:** Christen Rune Stensvold, Kateřina Jirků-Pomajbíková, Katrine Wegener Tams, Pikka Jokelainen, Rebecca P. K. D. Berg, Ellinor Marving, Randi Føns Petersen, Lee O’Brien Andersen, Øystein Angen, Henrik Vedel Nielsen

**Affiliations:** 1 Department of Bacteria, Parasites and Fungi, Statens Serum Institut, Artillerivej 5, DK-2300 Copenhagen S, Denmark; PIJO@ssi.dk (P.J.); rebe@ssi.dk (R.P.K.D.B.); rfp@ssi.dk (R.F.P.); obi@ssi.dk (L.O.A.); ysan@ssi.dk (Ø.A.); hvn@ssi.dk (H.V.N.); 2 Biology Centre, Czech Academy of Sciences, Institute of Parasitology, Branišovská 31, 370 05 České Budějovice, Czech Republic; pomajbikova@paru.cas.cz; 3 Department of Biotechnology and Biomedicine, Technical University of Denmark, Søltofts Plads, Bygning 221, DK-2800 Kongens Lyngby, Denmark; kawet@dtu.dk; 4 Department of Virus and Microbiological Special Diagnostics, Statens Serum Institut, Artillerivej 5, DK-2300 Copenhagen S, Denmark; EOR@ssi.dk

**Keywords:** metabarcoding, next-generation sequencing, zoonotic infections, parasite, parasitology, host specificity, genetic diversity, DNA, PCR

## Abstract

Several parasite species are shared between humans and pigs. We explored the application of next-generation sequencing-based metabarcoding supplemented with real-time PCR to fecal DNAs from 259 samples from 116 pigs in Denmark to detect and differentiate single-celled intestinal parasites of zoonotic relevance. *Enterocytozoon bieneusi*, *Balantioides coli*, and *Giardia duodenalis* were observed in 34/37 (92%), 148/259 (57%), and 86/259 (33%) samples, respectively. *Entamoeba polecki* ST1, *E. polecki* ST3, and *Entamoeba hartmanni* were detected in 104/259 (40%), 161/259 (62%), and 8/259 (3%) samples, respectively. Metabarcoding and real-time PCR detected *Cryptosporidium* in 90/259 (35%) and 239/259 (92%) of the samples, respectively, with *Cryptosporidium suis* and *Cryptosporidium scrofarum* observed in nearly equal proportions. *Blastocystis* subtypes 1, 3, 5, and 15 were found in 72 (28%), 6 (2%), 176 (68%), and 36 (14%) of 259 samples, respectively. *Iodamoeba* was identified in 1/259 samples (<1%), while none of 37 tested samples was positive for *Dientamoeba fragilis*. Our results illustrate how metabarcoding exemplifies a ‘one-fits-many’ approach to detecting intestinal single-celled parasites in feces supplemented with real-time PCR for selected parasites. Using metabarcoding with pathogen-specific assays may help detect emerging and previously underdetected pathogens and further elucidate the role of micro-eukaryotic parasites in human and animal health and disease.

## 1. Introduction

Several parasites can be hosted by both human and non-human hosts. Zoonotic intestinal parasitic genera taking a toll on both human and animal health include *Giardia* and *Cryptosporidium* [1]. Meanwhile, a few others can be observed with varying frequency in both humans and larger mammals, such as pigs, including *Enterocytozoon, Balantioides*, *Blastocystis*, *Dientamoeba*, and *Entamoeba* [2,3,4,5,6,7,8,9], the public health and animal health significance and epidemiology of which remain unclear.

The screening of human fecal samples for parasites of clinical and epidemiological relevance is increasingly being assisted by DNA-based tools such as targeted conventional and real-time PCRs [10] and commercial solutions offering multiplex PCR assays. Traditionally, routine parasitological testing of samples from domestic animals (including pigs) has relied mainly on coprological approaches using concentration techniques (e.g., flotation and sedimentation) [11], while molecular methods are increasingly used. The results from the current routine approaches often indicate only the presence/absence of parasites, with little or no information on genetic diversity (such as species, lineages, genotypes, subtypes, etc.) due to their high morphological uniformity (e.g., *Blastocystis* or *Giardia*) or due to the use of pathogen-specific targeted assays.

Meanwhile, next-generation sequencing (NGS)-assisted assays have been introduced as a comprehensive, ‘one-fits-many’ approach to detecting and differentiating parasites at a given taxonomic level. As an example, we previously introduced a metabarcoding assay relying on amplicon-based NGS of nuclear ribosomal genes from bacteria, fungi, and parasites with automated software-based annotation of sequences to genus and—oftentimes—species/sub-species level. This method has proven useful for screening various matrices such as skin samples, cornea scrapings, sewage samples, and human stool samples for parasites and other non-viral organisms, the relevance of which depends on the focus and scope of the investigation [12,13,14,15].

Based on available literature, shotgun sequencing was recently applied to a minor collection of fecal DNA from pigs for parasite detection and differentiation [16], and amplicon-based NGS of ribosomal DNA in fecal samples from pigs has been attempted once [17] with no reporting of, for example, *Giardia* and *Cryptosporidium*. The aim of this study was therefore to explore the applicability of the metabarcoding approach supplemented by specific real-time PCR tests for testing samples from pigs for intestinal parasitic protists, with a focus on protists that can be hosted by both pigs and humans. We discuss the benefits and challenges of the approach, and the results provide insight into the genetic diversity and host specificity of the detected genera.

## 2. Materials and Methods

### 2.1. Samples

A total of 273 fecal DNAs from 120 pigs were available from previous studies [18,19,20,21]. The 5–12 week-old pigs had been sampled in the weaning units of four conventional pig farms in different regions in Denmark [20]. Herd 1 (*n* = 34 samples) and Herd 5 (*n* = 33) samples were from Jutland, while Herd 3 (*n* = 28 samples) and Herd 4 (*n* = 178 samples) were from Zealand. The sampling procedure is described in the study by Græsbøll et al., 2017 [18], and the herd numbers applied in the present study are the same as in the study by Græsbøll et al. The samples were collected either at defecation or per rectum. A minor proportion (13%) of the pigs had received tetracycline prior to sampling (8/34 from Herd 1; 15/28 from Herd 3; 12/178 from Herd 5, and 0/33 from Herd 5). However, it was not a goal in this study to investigate the impact of tetracycline on parasite positivity.

### 2.2. DNA Extraction

All samples were subject to DNA extraction as previously described [19]. Briefly, total DNA was extracted using the Maxwell^®^ 16 LEV Blood DNA Kit (Promega Corporation, Madison, WI, USA). The samples were homogenized in a cell and tissue disruptor (Tissuelyser II, Qiagen, Hilden, Germany) with 5-mm stainless steel beads (Qiagen, Hilden, Germany) and bead-beaten for 2 min in a 25 mg/mL lysozyme buffer (Sigma-Aldrich, Søborg, Denmark A/S). Next, they were transferred to Maxwell^®^ extraction kit cartridges, and DNA was extracted according to manufacturer’s instructions. DNA concentration and purity were evaluated by the 260/280 nm-ratio using the NanoDropND-1000 spectrophotometer (NanoDrop Technologies Inc., Wilmington, DE, USA). The extracted DNAs were diluted to 40 ng/μL in nuclease-free water (Qiagen, Hilden, Germany) and stored at −20 °C until further processing.

### 2.3. Detection and Differentiation of Parasitic Genera by Metabarcoding

The DNAs were processed by the metabarcoding assay [12,13,14,15,22]. This method involves PCR-based amplification (PCR 1) of ribosomal genes using one set of primers targeting 16S and three sets of primers targeting 18S (Table 1). The G3 and G6 primers target the hyper-variable regions V3–V4 of the 18S, and G4 primers target V3–V5.

An adaptor PCR (PCR 2) was performed, and DNA concentration was quantified using the Quant-IT^TM^ High-Sensitivity dsDNA Assay Kit (Thermo Fisher Scientific, Hvidovre, Denmark). PCR2 products were pooled in equimolar amounts across samples. Undesirable DNA amplicons were removed from the pooled amplicon library (PAL) by Agencourt AMPure XP bead (Beckman Coulter) purification. The resulting AMPure beads-purified PAL (bPAL) was diluted to its final concentration of 11.5 pM DNA in 0.001 N NaOH and used for sequencing on the Illumina MiSeq desktop sequencer (Illumina Inc., San Diego, CA, USA). The library was sequenced with the 500-cycle MiSeq Reagent Kit V2 in a 2 × 250 nt setup (Illumina Inc., San Diego, CA, USA).

Raw reads were assigned to taxon by the “BION” package (http://box.com/bion, accessed on 28 May, 2021), and involved quality trimming, read pairing, and chimera filtering before taxonomic classification of sequences. Sequences from the three 18s rRNA targets were queried against the SILVA database in combination with an in-house database.

### 2.4. Real-Time PCR for Cryptosporidium, Dientamoeba, Enterocytozoon, and Giardia

Based on previous experience [15], the sensitivity of the metabarcoding assay in terms of detecting *Giardia*, *Dientamoeba,* and microsporidia was expected to be low; therefore, specific molecular assays were applied for these.

We applied the duplex real-time PCR for *Cryptosporidium* spp. and *Giardia duodenalis* that is used in the Laboratory of Parasitology, Statens Serum Institut to all the DNAs. The method has been applied in One Health context previously [23], and the primers used are listed in Table 2. This was done to ensure detection of *Giardia*, and to add support to the data output on *Cryptosporidium* generated by metabarcoding.

The 25-µL reaction mixture contained 0.2 µL IMMOLASE™ DNA Polymerase (Bioline, Denmark), 2.5 µL 10 × ImmoBuffer (Bioline), 2.5 µL 10× dUTP (1×), 2.5 µL 50 mM MgCl2 (5 mM), 0.5 µL ROX Reference Dye (Invitrogen) (diluted 1:30), 5 µL 50% glycerol (5%), 1.25 µL (1 µM) of each primer, 0.125 µL (0.075 µM) of each probe, 0,25 µL IPC (Internal Process Control DNA), 5 µL of DNA eluate, and water sufficient to reach the total volume of 25 µL. Negative (water) and positive (*G. duodenalis* and *C. parvum* DNA) controls and inhibition controls were included in each run on the Applied Biosystems QuantStudio™ 5 Real-Time PCR System (Thermo Fisher Scientific, Hvidovre, Denmark). PCR cycling conditions were as follows: 10 min at 95 °C (initial denaturation), 50 cycles of 15 s at 95 °C, and 60 s at 60 °C. PCR products were analyzed with the QuantStudioTM Design&Analysis Software v1.5.1 (Thermo Fisher Scientific, Hvidovre, Denmark). Samples were considered positive, if they exhibited a sigmoid function with a threshold cycle value (Ct-value) ≤ 42.

Similarly, we screened a subset of the samples (n = 37; chosen by convenience sampling) for *Dientamoeba fragilis* [24] and *Enterocytozoon bieneusi* [25] using the same PCR conditions as described above. The primers used are listed in Table 2.

Conventional PCR followed by Sanger sequencing was applied to a minor selection of samples that were real-time PCR-positive but metabarcoding-negative for *Cryptosporidium* for test result confirmation; the primers published by Xiao et al. [26] were used.

Genotyping of *E. bieneusi* was performed using the method by Buckholt et al., 2002 [27].

**Table 2:** Oligos used in the present study for real-time PCR-based detection of selected parasites.

Parasite (Target Gene)	Oligonucleotides (Primer/Probe)	Primer and Probe Sequences	PCR Product Size (bp)	Reference
*Cryptosporidium* spp.	Cryptosporidium CRY F3	5′-CTA CAC TGA TGC ATC CAT CRA GT-3′	78	Present study
(18S)	Cryptosporidium CRY R3	5′-CCC ATC ACG ATG CAT AYT CAA AA-3′		
	Cryptosporidium CRY P	VIC-TCC TGT TTC GAA GGA AAT GGG TAA TC-MGB		
*Dientamoeba fragilis*	DF-124f	5′-CAA CGG ATG TCT TGG CTC TTT A-3′	97	[24]
(ITS1-5.8S-ITS2)	Df-221r	5′-TGC ATT CAA AGA TCG AAC TTA TCA C-3′		
	Probe Df-172	6-Fam CAA TTC TAG CCG CTT AT-MGBNFQ		
*Enterocytozoon bieneusi*	EblTS-89F	5′-TGT GTA GGC GTG AGA GTG TAT CTG-3′	103	[25]
(ITS)	EblTS-191r	5′-CAT CCA ACC ATC ACG TAC CAA TC-3′		
	Probe EblTS-114rev T	FAM-CAC TGC ACC CAC ATC CCT CAC CCT T-BHQ-1		
*Giardia duodenalis*	Giardia-80F	5′-GAC GGC TCA GGA CAA CGG TT-3′	62	[28]
(18S)	Giardia-127R	5′-TTG CCA GCG GTG TCC G-3′		
	Giardia-105T	FAM-CCC GCG GCG GTC CCT GCT AG-BHQ-1		

### 2.5. Analysis of DNA Sequence Read Outputs

Samples with 1000 reads or fewer mapping to eukaryotes were excluded from the analysis, as such low read counts most likely reflected PCR inhibition and would not reflect the true distribution of eukaryotic organisms in the sample.

The highest sequence read output for *Cryptosporidium* was obtained by the G3 primers; for *Balantioides*, the sequence output generated by the G4 primer pair was used (Table 3). For *Blastocystis*, *Entamoeba,* and *Iodamoeba*, the G6 primer pair produced the largest sequence read output.

For each sample positive for parasites (except for *Balantioides* and *Blastocystis* ST15 for which a different approach was used), DNA sequences were downloaded in fasta format for each relevant genus from the BION server and collated in sample-specific files. DNA sequences were submitted to multiple sequence alignment using Clustal Omega (https://www.ebi.ac.uk/Tools/msa/clustalo/; accessed on 28 May, 2021), and each alignment was inspected by eye to identify sequence variation that could indicate genetic diversity and not PCR or sequencing error. For each major cluster of near-identical sequences (sporadic single nucleotide polymorphism [SNPs] were commonly noticed and thought to be due to PCR and sequencing-introduced errors), a consensus sequence was generated, recorded, and queried in the NCBI Database (https://blast.ncbi.nlm.nih.gov/Blast.cgi; accessed on 28 May 2021). Sequences are available at GITHUB (https://github.com/Entamoeba/pig-study-2020; accessed on 28 May 2021). Specifically, for *Balantioides*, sequence reads from a variety of positive samples were pooled with a view to identifying major lineages. This approach was applied to *Balantioides* because of (1) a large amount of minor genetic diversity per sample and (2) the fact that most of the sequences had gaps in the middle (the PCR product generated by the G4 primers is larger than those generated for the G3 and G6 primers, and sequencing using ILLUMINA may not provide sufficient coverage over the middle part of the amplified fragment). For sequences reflecting *Blastocystis* ST15, examples of sequences were pooled and uploaded as documentation (GITHUB). It should be noted that for those samples potentially positive for ST15, the number of ST15-associated reads was limited (typically between 1 and 50). Even the pooled sequences did not align very well and none of the consensus sequences that could be generated were 100% similar to sequences in GenBank. 

For *Balantioides*, *Blastocystis*, and *Entamoeba*, samples for which <40 sequence reads were observed per genus were deemed negative for that particular genus. This arbitrary threshold was chosen in order to avoid erroneous classification of a sample with very few sequence reads as positive, since these sequence reads might reflect spill-over of ID tags from highly positive samples. Since the average number of *Cryptosporidium*-specific reads was generally noticeably lower in comparison, samples with *Cryptosporidium*-specific sequence reads >0 were scored as positive.

## 3. Results

### 3.1. DNA Sequence Read Yield in the Metabarcoding Assay

The total number of sequence reads obtained by all primer sets that could be mapped was 19,777,284 (Table 4), with a median combined number of Archaea, Prokaryotes, and Eukaryotes reads of 72,180 per sample (interquartile range, 50,922–87,416). Fourteen of the samples (5%) had very few reads representing eukaryotic DNA (~1000 reads or fewer, indicating PCR inhibition), so these samples were excluded from the sample set. Hence, the overall number of samples included for data analysis was 259 from 116 pigs.

Data on DNA sequence read yield obtained per genus by the metabarcoding assay are also listed in Table 4.

### 3.2. Parasites Detected by Metabarcoding and Real-Time PCR

The genera of potential zoonotic relevance detected by metabarcoding were *Balantioides*, *Blastocystis*, *Cryptosporidium*, *Entamoeba*, and *Iodamoeba*. Meanwhile, *Dientamoeba*, *Enterocytozoon*, and *Giardia* were not detected by the metabarcoding assay in any of the samples (Table 4), supporting the low sensitivity of the assay for these genera.

With regard to real-time PCR results, 86 (33%) samples tested positive for *Giardia.* Meanwhile, the number of samples positive for *Cryptosporidium* was 239 (92%), which was a substantially higher positivity rate than that obtained by the metabarcoding assay (35%). The real-time PCR cycle threshold values for the *Cryptosporidium*-positive samples ranged from 22 to 40, with a median (interquartile range [IQR]) of 32 (30–34). Given this IQR, most of the samples identified as positive by the real-time PCR assay could be considered weakly positive samples. For the 113 samples that had a Ct value ≥ 33, 15 (13%) were positive by the metabarcoding assay. Meanwhile, for the 126 samples with a Ct value < 33, 72 (57%) were identified as *Cryptosporidium*-positive by metabarcoding; these observations clearly support a higher sensitivity of the real-time PCR assay. Six samples with Ct values < 30 that were negative by the metabarcoding assay were subjected to conventional PCR followed by Sanger sequencing. In all six cases, sequencing of the PCR products revealed *C. scrofarum*.

While none of the subset samples were positive for *D. fragilis*, 34 (92%) of the 37 samples were positive for *E. bieneusi*. One genotype of *E. bieneusi* was observed by nested PCR and Sanger sequencing, namely EbpA.

**Figure 1:**
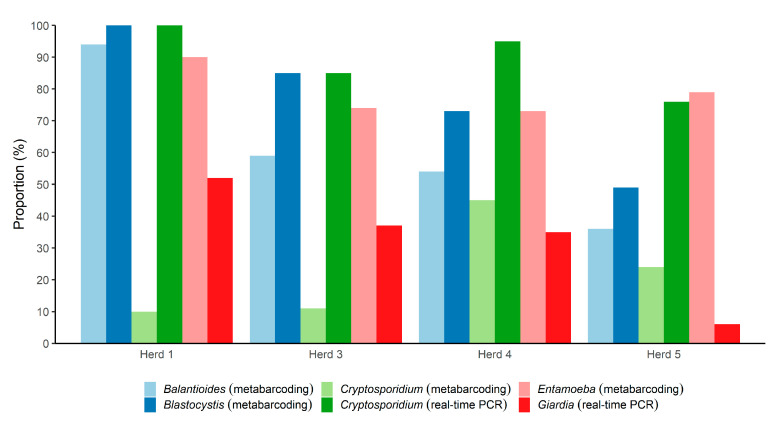
Overview of the proportions of the 259 fecal DNAs from pigs from four different herds in Denmark positive for five parasitic genera, according to herd. For *Balantioides*, *Blastocystis*, and *Entamoeba*, only metabarcoding was used. For *Giardia* and *Cryptosporidium*, both metabarcoding and real-time PCR were used (*Giardia* failed to be detected by the metabarcoding assay).

An overview of the parasite positivity rate according to herd is provided in Figure 1. Comparing the herds, it appeared that the positivity rate was generally lower in Herd 5 than in the other three herds, whereas samples from Herd 1 exhibited very high positivity rates overall. In samples from Herd 4, *Cryptosporidium* was quite common compared with the other herds as observed by the microbiome assay (Figure 1); however, real-time data revealed that the positivity rate was high across all herds. For *Giardia*, the situation was different, with 6% of samples positive from Herd 5, compared with 47% in samples from Herd 1.

### 3.3. Differentiation of Species and Subtypes Detected in the Samples by Metabarcoding

A detailed presentation of the species and subtypes identified in the samples by metabarcoding is provided in Table 5. The sequences obtained for the organisms below are available on GITHUB (https://github.com/Entamoeba/pig-study-2020; accessed on 28 May 2021).

#### 3.3.1. *Cryptosporidium*

By metabarcoding, *C. suis* and *C. scrofarum* were identified in 54/259 (21%) and 46/259 (18%) of the samples, respectively; no other species of *Cryptosporidium* were observed. Eight samples were positive for both *C. suis* and *C. scrofarum.*

No intra-species DNA sequence variation was observed for *Cryptosporidium*; all *C. suis* sequences were identical to AF108861 [29], while all *C. scrofarum* sequences were identical to e.g., JX424840 [30]. All samples with *Cryptosporidium*-specific reads were positive for *Cryptosporidium* by real-time PCR. Overall, the Ct values of the *C. suis*-positive samples were lower than those of the *C. scrofarum-*positive samples. Meanwhile, the crude read counts generated by the metabarcoding assay appeared similar for the two species.

#### 3.3.2. *Giardia*

The metabarcoding assay did not detect *Giardia* in any of the samples, while the real-time PCR-based analysis of the samples identified *Giardia* in 86 samples (33%).

#### 3.3.3. *Entamoeba*

About three out of four samples were positive for species of *Entamoeba*, in particular *E. polecki*, which was found in 193 samples (75%). *E. polecki* ST1 was detected in 104 samples (40%), while *E. polecki* ST3 was found in 161 samples (62%). Both subtypes were observed in 37.4% of the *E. polecki*-positive samples. No intra-subtype diversity was observed in any of the two subtypes.

*E. hartmanni* was detected in 8 (3%) samples, and all *E. hartmanni* consensus sequences exhibited 100% similarity to GenBank accession numbers FR686375 and FR686374, which are sequences of *E. hartmanni* found in human stool in Sweden [31].

#### 3.3.4. *Iodamoeba*

Among the 259 samples, one tested positive for *Iodamoeba bütschlii*. The results for the two remaining primer sets (G3 and G4) were also checked for *Iodamoeba*-specific sequences; however, no evidence of other positive samples was observed. The file with the sequence output exemplifies the extreme variability seen among *Iodamoeba*-specific SSU rRNA genes that can be present in a fecal sample, and the consensus sequence ’731IOa_RL2′ (https://github.com/Entamoeba/pig-study-2020; accessed on 28 May, 2021) was generated from only a few of the reads and showed 100% similarity to *Iodamoeba* sp. RL2.

#### 3.3.5. *Balantioides*

*Balantioides* was detected in 57% of the samples. The positivity rate of the parasite differed substantially across the herds (Figure 1). While a positivity rate of 93.5% was observed among samples from Herd 1, it was 36.4% in samples from Herd 5; the positivity rates in samples from Herd 3 and Herd 4 were 59% and 54%, respectively.

Two different sequence types were identified: One had a 100% match to MK801486 from a pig sampled in Germany [16] and 99.79% to several DNA sequences from domestic pigs sampled in the Czech republic (JQ07324,-23,-21-04) and in Cameroon (JQ07334) [4]; we refer to this sequence as ‘sequence type I’ and it was present in 64.2% of the *Balantioides*-positive samples. The other sequence type (‘sequence type II’) shared 100% identify to, for example, MK801495 from a pig in Germany [16] and GQ903678 from a pig sampled in the Philippines [32] and present in the remaining *Balantioides*-positive samples (35.8%).

#### 3.3.6. *Blastocystis*

**Figure 2:**
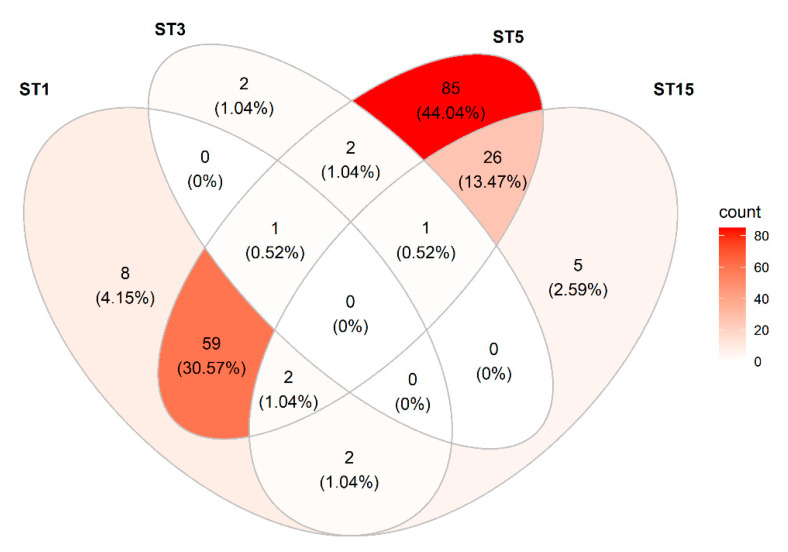
Venn diagram showing the distribution and co-occurrence of *Blastocystis* sp. subtypes detected in the study of fecal DNAs from pigs from four different herds in Denmark.

*Blastocystis* was observed in 193/259 (75%) samples. Sixty-eight percent of all tested samples were positive for ST5 (Table 5), and ST5 was observed in 91% of all *Blastocystis*-positive samples (Figure 2). ST1 was seen in 28% of the samples, ST15 in 14%, and ST3 in 2% of the samples. ST15 was typically observed admixed with ST5 (81% of the ST15-positive samples were positive for ST5) (Figure 2). Both ST1 and ST5 were seen in 62 samples (32% of all *Blastocystis*-positive samples); both ST1 and ST15 were observed in 29 samples (15% of all *Blastocystis*-positive samples), and both subtypes 1 and 3 were seen in one sample (0.5% of all *Blastocystis*-positive samples). One sample was found to be positive for subtypes 1, 3, and 5, and another sample was positive for subtypes, 3, 5, and 15.

All samples from Herd 1 were positive for *Blastocystis* sp. ST5, and some were positive for other subtypes as well: typically, ST1, but also a few ST15.

For ST5, several different sequence types were observed (ST5a through ST5f; Table 6), although ST5a and ST5c were strikingly similar and maybe the same, and this was also the case for ST5d and ST5f; these variants differed by only one or two SNPs. Similarly, for ST1 and ST3, two different sequence types were observed for each (Table 6).

For ST3, two different sequence types were observed; one sample was positive for both types, while the other ST3-positive samples had only one of the two types.

### 3.4. Examples of Detected Polyparasitism

Most samples tested positive for multiple parasitic species. For instance, 164 of 193 (85.0%) of the *Blastocystis*-positive samples were positive for *E. polecki*, and 108 of these 164 *Blastocystis*- and *Entamoeba*-positive samples (65.9%) were positive for *Balantioides*. One hundred and six (41%) of the samples were positive for *Balantioides*, *Blastocystis*, and *Entamoeba*. Forty-four (17%) samples were positive for *Cryptosporidium*, *Giardia, Balantioides*, *Blastocystis*, and *Entamoeba*.

Of the 54 *C. suis*-positive samples, 40 (74.1%) and 37 (68.5%) were positive for *E. polecki* and *Blastocystis*, respectively, while 36 (78.3%) and 40 (87.0%) of the 46 *C. scrofarum*-positive samples were positive for the two, respectively.

The single sample in which *Iodamoeba* was observed was also positive for *C. suis*, *C. scrofarum*, *E. polecki* ST1, *E. polecki* ST3, *Blastocystis* ST5, and *Blastocystis* ST15.

## 4. Discussion

The amplicon-based NGS-based approach to detecting and differentiating parasites used in this study has been applied in a number of studies involving human, animal, and environmental samples, including corneal scrapings, fecal samples, and sewage samples [14,15,22,33], and with different foci from clinically relevant opportunistic parasites to foodborne parasites [34]. This ‘one-fits-many’ approach could be cost-effective for screening DNAs from large numbers of samples for DNA from parasites, fungi, and bacteria, although some limitations have been identified [15,34].

In the present study, we screened for intestinal protists previously reported in pigs, focusing on those with zoonotic potential. Of the species detected, *E. hartmanni*, *Blastocystis* sp. ST1 and ST3, and *I. bütschlii* are all common in humans. *E. polecki* is rarely reported in humans [35], and the same is true for *Blastocystis* sp. ST5 (see below), *C. suis* [36], and *Balantioides coli* [6], although the distribution of reported human cases of balantidiosis differs substantially according to geographical region [6]. *Cryptosporidium scrofarum*, previously referred to as *Cryptosporidium* pig genotype II, has been reported in at least one human case [37].

Our metabarcoding approach was supplemented by real-time PCR for *Dientamoeba*, *Enterocytozoon*, *Giardia,* and *Cryptosporidium* in order to increase test sensitivity for these parasites. Interestingly, *Giardia* was not identified in any of the pig samples subject to shotgun sequencing by Wylezich et al. [16]. That team recently published an evaluation of their method [38] and mentioned the low recovery rate for *Giardia* as a limitation, which means that *Giardia* may have been overlooked in their study of pigs [16], and thus, it is worth noticing that *Giardia* may generally be difficult to detect not only by the metabarcoding methods but also by a metagenomics approach. To this end, Ramayo-Caldas et al. [17] used amplicon-based NGS relying on detection and differentiation of ITS and 18S rRNA fragments and mentioned neither *Giardia* nor *Cryptosporidium* among their findings. In the study by Parfrey and colleagues [39], one set of primers was used to amplify eukaryotic DNA in human stool samples, and also here, *Giardia*- and *Cryptosporidium*-specific DNA was not detected. The authors speculated that the primers might not be suited for the detection of *Giardia* and that DNA extraction might not have been optimal for the detection of *Cryptosporidium*. However, there was no baseline or reference data (e.g., microscopy data) indicating whether any of the samples included in that study were positive for these two parasites.

Although this study was not designed to evaluate the sensitivity of the metabarcoding assay, it is worth noting that the sensitivity of the assay appears to differ according to genus. While *Enterocytozoon* and *Giardia* were not detected at all by the metabarcoding assay, *Cryptosporidium* was identified in some samples but to a much lesser extent than by real-time PCR. Interestingly, *Cryptosporidium* was readily detected in samples with *Cryptosporidium* amplicon-specific Ct values lower than 33. The reason for the relatively high positivity rate in samples from Herd 4 by the metabarcoding assay might reflect the fact that 54/71 (76%) samples with *Cryptosporidium*-specific Ct values by real-time between 22 and 30 were from this herd, and so these would be more likely to contain sufficient *Cryptosporidium*-specific DNA detectable by the metabarcoding assay. We successfully sequenced *Cryptosporidium*-specific SSU rDNA from all six samples that were real-time PCR-positive with Ct values < 30 and metabarcoding-negative, which confirms the higher sensitivity of the real-time PCR assay compared with the metabarcoding assay.

*Cryptosporidium parvum*, which has previously been reported in pigs in Sweden [40] but not in Denmark [41], was not detected in the present study.

The Ct values of the *C. suis*-positive samples were significantly lower than those of the *C. scrofarum*-positive samples. This may reflect (i) a higher infection intensity for *C. suis* than for *C. scrofarum*, (ii) a difference between the two species in terms of oocyst resistance towards DNA extraction, (iii) genetic polymorphism in the primer-probe annealing sites, resulting in preferential amplification/probe-based detection of *C. suis* over the *C. scrofarum*, and/or (iv) variation in copy number of the SSU rRNA gene between these two species. Of these scenarios, the first one might be of particular relevance here, since an earlier study from Denmark showed that pigs infected with *Cryptosporidium scrofarum* excreted fewer oocysts compared with pigs infected with *Cryptosporidium suis* [42], adding support to the observations in the present study.

*Enterocytozoon bieneusi* is a common finding in pigs all over the world, and multiple genotypes have been observed in this host type [9], some of which are zoonotic. For example, Sak and colleagues [43] detected *E. bieneusi* in 74/79 pigs (94%) in the Czech Republic. In that study, nested PCR was used for screening, and most of the *E. bieneusi*-positive pigs (70/74, 95%) had the zoonotic genotype EbpA, which is the one identified in the present study. In the present study, only EbpA was identified, and although we only tested a minor subset of samples, the subset included samples from all four pig herds, and thus our finding suggests that this may be a common genotype in pig herds in Denmark. *Enterocytozoon bieneusi* has been a rare observation in human fecal samples in Denmark. However, it should be mentioned that there was an *E. bieneusi* outbreak in 2020 in a company based in the Copenhagen metropolitan area (Michlmayr et al., in preparation); in this outbreak, Genotype C was identified. The same genotype was also the cause of an outbreak in Sweden in 2009 [44]. To date, no evidence of an animal reservoir for Genotype C has been identified [45].

*Dientamoeba fragilis* is very common in the human population in Denmark [46,47]. Data from Italy suggested that pigs may be common and natural hosts of *D. fragilis* [5,48]. Since we did not detect this parasite in any of the samples, we were not able to produce evidence supporting those findings.

Other parasites (*Blastocystis*, *Entamoeba*, and *Balantioides*) were detected in most samples, and the current version of the metabarcoding assay therefore appears to be particularly useful for detecting these.

*Blastocystis* is very common in the human population, including in Denmark [49,50]. The observation that *Blastocystis* sp. ST1 is so common in samples from pigs from Denmark suggests a potential for zoonotic transmission, given the fact that ST1 is one of the most common subtypes found in the human population in Denmark [49,50]. A previous study on *Blastocystis* in pigs in Denmark revealed only ST5 and ST3, with a clear preponderance of ST5 [51]. Meanwhile, Navarro et al. found ST1 to predominate among pigs sampled in Spain [52], and ST1 has also been found to be common in pigs in Thailand [53] and goats in Malaysia [54]. Apart from ST1, ST5 appears to be the most common subtype in domestic pigs [55,56], and this subtype has only rarely been observed in human fecal samples [57]. It should be noted, however, that the two sequence types displayed in Table 6 would both be scored as ST1 allele 4 according to the terminology by Stensvold et al. [58], and so the allele system, which is currently commonly used, might not be sufficiently discriminative to separate ST1 strains of uncertain host specificity. *Blastocystis* ST15 was described for the first time in 2013 by Alfellani et al. [59] in a camel and a gibbon. Since then, this subtype was found to be common in wild boars sampled in Italy and it was also found in a couple of domestic pigs [60]. In the study by Wylezich et al. [16], 14/41 samples were positive for ST15. Hence, just like ST5, ST15 appears to be a parasite of mainly suids.

The intra-subtype variation accounted for in Table 6 shows the extent of within-subtype variation in *Blastocystis* and the discriminatory ability of the metabarcoding assay.

Of particular note is the finding of *E. hartmanni*, which has not previously been reported in pigs. Kessel reported *Entamoeba* cysts with 1–4 nuclei in pigs, and the cysts were between 5 and 12 µm, which would include the *E. hartmanni* cyst size range. These were referred to as *E. dysenteriae*-like, which is a synonym of *E. histolytica*, and at that time, *E. hartmanni* was referred to as ‘small-race *E. histolytica*’. Burrows indicated that *E. histolytica* might frequently have been mixed up with *E. hartmanni* [61,62]. In a study specifically investigating the distribution of *Entamoeba* spp. in pigs in China by DNA-based methods, Li et al. tested for *E. histolytica*, *E. suis*, and *E. polecki*, but not for *E. hartmanni*. No cases of *E. histolytica* were found [63].

*E. polecki* was divided into four specific subtypes by Verweij et al. in 2001 [64], all of which have been isolated from human stool, but which appear to differ in terms of host reservoir specificity. Of these four subtypes, ST1 and ST3 appear to be common in pigs [16,63,65,66]. Meanwhile ST4 has only been found in humans so far, whereas ST2 has been found in non-human primates as well. In the present study, subtypes 1 and 3 were found in 41% and 63% of the samples, respectively, and none of the remaining two *E. polecki* subtypes were found. It should be noted that even though both subtypes of *E. polecki* appear to be very common in pigs on a general basis and even though these subtypes may be found in human stool samples, the extent of human infection/colonization appears limited [35].

Of note, there was no evidence of *Entamoeba suis*-specific DNA in the samples. *E. suis* was observed in 81.1% samples from pigs sampled in West Java, Indonesia [66], while Ji et al. observed a considerable lower positive rate (13.0%) for *E. suis* in pigs sampled in south-eastern China [65]. To our knowledge, *E. suis* is yet to be reported and confirmed at DNA-level in Europe.

The zoonotic ciliate *Balantioides coli* was detected in a total of 57% of samples in this study, with the proportion of positives varying between herds and ranging between 36.4% and 93.5%. This is consistent with the results from prevalence studies from other countries [16,17,67,68]. In the present study, based on the SSU rDNA marker, two types of *B. coli* sequences were detected, for which we introduced a designation: sequence types I and II. Similar results were revealed in the previous molecular-phylogenetic study focused on the genetic diversity of *B. coli* in pigs and primates [4], in which two clusters of *B. coli* were revealed using SSU rDNA sequence analysis; one group corresponding to sequence type I showed a broader host specificity and included strains from domestic pigs from different localities (Philippines, Spain, Central African Republic, Czech Republic, and Kenya), wild boar (Czech Republic), and captive apes (chimpanzees and gorillas from different zoological gardens and rehabilitation centers). The second group, which corresponds to sequence type II was dominated by *B. coli* sequences from domestic pigs sampled in Czech Republic, Madagascar, and Cameroon. Both sequence variants of *B. coli* were detected in a later large-scale study in domestic pigs in Germany [16]. Unfortunately, these data cannot be compared with data from other studies on molecular detection of *B. coli* and epidemiology because they address its genetic diversity using hypervariable marker ITS1-5.8rRNA-ITS2 [69,70]. Although several variants of *B. coli* (A0–A2, B0, B1) have been described based on the primary and secondary structure of the ITS markers, it is unclear whether this may suggest host specificity or transmission due to the presence of more variants in one isolate, even within a single *B. coli* cell, also in different host species [71]. Despite this, Pomajbíková et al. [4] showed that two clusters based on the SSU sequences in *B. coli* (corresponding to the two sequence types I and II in this study) were supported also by ITS DNA sequence analysis. Our present results suggest that the SSU rDNA marker may be sensitive enough to distinguish intraspecific variability of *B. coli*, and information on sequence types might assist in studies of zoonotic transmission, especially studies involving pig farms or slaughterhouses and the personnel in these places. A recent study from Argentina confirmed 100% genetic identity between isolates obtained from pigs and humans using the SSU marker, which may suggest zoonotic transmission [72].

It might be surprising that only one sample was positive for *Iodamoeba*. In the study by Wylezich et al. [16], 13/41 (32%) pigs tested positive for *Iodamoeba*, and the authors identified RL2 in all the *Iodamoeba*-positive samples. RL2 has previously been identified in pig feces as well as in human stool [73]. *Iodamoeba*-specific PCR could be used to confirm this relatively low positivity rate.

A number of pigs received tetracycline treatment prior to the sampling. However, our aim was not to investigate the effect of tetracycline on parasite positivity. Suffice to say that since 13% of the pigs had received antibiotics prior to sampling, this might to a minor extent account for one or more of the differences observed in parasite positivity rate.

Using NGS-based metabarcoding in addition to pathogen-specific assays may be useful for detection of emerging and previously underdetected pathogens in a One Health setting, across host species. We observed extensive examples of polyparasitism in this study, which highlights the relevance of using assays such as the metabarcoding assay used in the present study, not only when the aim is to screen for multiple parasites but also when multiple taxa (species, subtypes) of parasites are expected. It should be noted that little data is available with regard to the limit of detection of the metabarcoding assay for most of the parasites studied here. Meanwhile, the assay appears to be a suitable method for generating baseline data on parasite occurrence and diversity in various hosts.

## Figures and Tables

**Table 1:** Primers used in the metabarcoding assay.

Primer Pair	Small Subunit rDNA Target	Primer Sequences
341F3/806R5	16S	5′-ACTCCTAYGGGRBGCASCAG-3′ 5′-AGCGTGGACTACNNGGGTATCTAAT-3′
G3F1/G3R1	18S	5′-GCCAGCAGCCGCGGTAATTC-3′ 5′-ACATTCTTGGCAAATGCTTTCGCAG-3′
G4F3/G4R3	18S	5′-CAGCCGCGGTAATTCCAGCTC-3′ 5′-GGTGGTGCCCTTCCGTCAAT-3′
G6F1/G6R1	18S	5′-TGGAGGGCAAGTCTGGTGCC-3′ 5′-ACGGTATCTGATCGTCTTCGATCCC-3′

**Table 3:** DNA sequence read yield obtained per genus by metabarcoding of 259 fecal DNAs from pigs from four different herds in Denmark.

Genus	Primer Set *	Sequence Reads per Sample, Range	Sequence Reads per Positive Sample, Median (IQR)	No. of Samples Positive/Tested (%)	No. of Samples Positive/Tested by Real-Time PCR
*Balantioides*	G4	0–41,925	508 (215.5–1404.5)	148/259 (57%)	NA
*Blastocystis*	G6	0–19,318	681 (212.5–2439.5)	193/259 (75%)	NA
*Cryptosporidium*	G3	0–4678	560 (169–1096)	90/259 (35%)	239/259 (92%)
*Dientamoeba*	NA	NA	NA	NA	0/37 (0%)
*Entamoeba*	G6	0–37,557	602 (225–2361)	195/259 (75%)	NA
*Enterocytozoon*	NA	NA	NA	NA	34/37 (92%)
*Giardia*	NA	NA	NA	NA	86/259 (33%)
*Iodamoeba*	G6	NA	NA	1/259 (<1%)	NA

* see text for details. NA = not applicable.

**Table 4:** Total number of DNA sequence reads generated by the metabarcoding assay according to taxonomic group across all primers sets.

	Number of Reads
TOTAL	19,777,284
Archaea	262,016
Prokaryotes	11,406,189
Eukaryotes	8,109,079
*Blastocystis*	1,568,032
*Entamoeba*	396,129
*Balantioides*	265,409
*Cryptosporidium*	80,159
*Iodamoeba*	1273
*Enterocytozoon*	0
*Giardia*	0
*Dientamoeba*	0
Fungal DNA	2,239,641
Host DNA	1,357,652
Plant DNA	1,236,282
Other (e.g., nematodes)	964,502

**Table 5:** Parasitic species and subtypes of *Balantioides*, *Cryptosporidium*, *Entamoeba*, and *Blastocystis* identified by cluster analysis of metabarcoding data obtained from 259 fecal DNAs from pigs from four different herds in Denmark.

Genus	Species	Subtype	No. of Samples Positive (%)
*Blastocystis*	sp.	ST1	72 (28%)
	sp.	ST3	6 (2%)
	sp.	ST5	176 (68%)
	sp.	ST15 *	36 (14%)
*Entamoeba*	*hartmanni*	NA	8 (3%)
	*polecki*	ST1	104 (40%)
	*polecki*	ST3	161 (62%)
*Iodamoeba*	*bütschlii*	RL2	1 (0.4%)
*Cryptosporidium*	*suis*	NA	53 (20%)
	*scrofarum*	NA	45 (17%)
*Balantioides*	*coli*	NA	148 (57%)

NA = not applicable; * Sequence data for all samples scored by BION as positive for ST15 were pooled and a consensus sequence was made based on the longest sequences.

**Table 6:** Sequence types of *Blastocystis* subtypes 1, 3, 5, and 15 observed in the present study.

Subtype	Sequence Type	Number of Samples Positive	Examples of Closest Matches to Reference DNA Sequence Entry (Accession no.) in NCBI Database
ST1	ST1a	49	AB107961 (pig, Japan) (99.76%)
	ST1b	55	AB107962 (human, Japan) (100%) KU719525 (human, Iran) (100%) MK801400 (pig, Germany) (100%)
ST3	ST3a	3	AB070986 (human, Japan) (100%) MT330276 (human, Thailand) (100%) AM779042 (human, Turkey) (100%) MK801389 (pig, Germany) (100%)
	ST3b	5	KM216257 (human, Thailand) (100%) LC414152 (human, Iran) (100%) MK418914 (macaque, China) (100%)
ST5	ST5a	2	AB107966 (cattle, Japan) (99.76%) AB070998 (pig, Japan) (99.76%) MK801415 (pig, Austria) (99.76%)
	ST5b	60	AB107964 (pig, Japan) (100%) MH104976 (pig, Thailand) (100%) MK801372 (pig, Germany) (100%)
	ST5c	8	AB107966 (cattle, Japan) (100%) AB070998 (pig, Japan) (100%) MK801415 (pig, Austria) (100%)
	ST5d	8	MK375237 (human, Thailand) (99.76%) MK801375 (pig, Germany) (99.76%) MK375227 (pig, China) (99.76%)
	ST5e	27	KT819615 (pig, Thailand) (100%) MK375229 (pig, China) (110%)
	ST5f	129	MK375237 (pig, China) (100%) MK801375 (pig, Germany) (100%)
ST15	ST15*	NA *	MK801393 (pig, Germany) (99.34%) KC148211 (gibbon, zoo) (99.12%) KC148210 (camel, Egypt) (97.81%)

* = due to the low number of sequence reads per positive samples, sequence reads were pooled and one consensus sequence was generated from those few sequences that did not exhibit any gaps in the middle. NA = not applicable.

## Data Availability

Data are available at https://github.com/Entamoeba/pig-study-2020 (accessed on 28 May 2021).
